# An ESIPT based naphthalimide chemosensor for visualizing endogenous ONOO^−^ in living cells†

**DOI:** 10.1039/c7ra11774d

**Published:** 2018-01-08

**Authors:** Yunshuang Fu, Hailiang Nie, Rubo Zhang, Fangyun Xin, Yong Tian, Jing Jing, Xiaoling Zhang

**Affiliations:** Key Laboratory of Cluster Science of Ministry of Education, Beijing Key Laboratory of Photoelectronic/Electrophotonic Conversion Materials, Analytical and Testing Center, School of Chemistry and Chemical Engineering, Beijing Institute of Technology 5 Zhongguancun Road Beijing 100081 P. R. China zhangxl@bit.edu.cn hellojane@bit.edu.cn +86-10-88875298

## Abstract

Based on ESIPT, we designed and synthesized a naphthalimide chemosensor N-CBT for selectively visualizing endo/exogenous peroxynitrite (ONOO^−^) in living cells. The incorporation of 2-benzothiazoleacetonitrile offers N-CBT a rare pre-existing eight-membered ring hydrogen bonding configuration, which is able to generate two types of emission of naphthalimide. Confirmed by calculation results, fast proton transfer from the hydroxyl group to the carbonyl group occurs along with excited-state energy transfer *via* the intramolecular H-bond, leading to a tautomeric transformation from the excited enol form to the excited keto form. In aqueous solution, the formation of intermolecular hydrogen bonding with water perturbs ESIPT and destroys the stable planar construction. By breaking the cyano carbon–carbon double bond in the presence of ONOO^−^, green fluorescence can be regenerated efficiently. As a result, 34-fold fluorescence enhancement at 518 nm was observed in response, and it showed a good linear relationship in the range of 1 to 14 μM with a detection limit of 37 nM. Subsequently, N-CBT was applied in visualizing cellular ONOO^−^, and it demonstrated great potential in selectively visualizing endo/exogenous peroxynitrite (ONOO^−^) in living cells.

## Abstract 2

An ESIPT based naphthalimide chemosensor with high sensitivity and selectivity for visualizing endogenous ONOO^−^ in living cells was developed.

## Graphical abstract

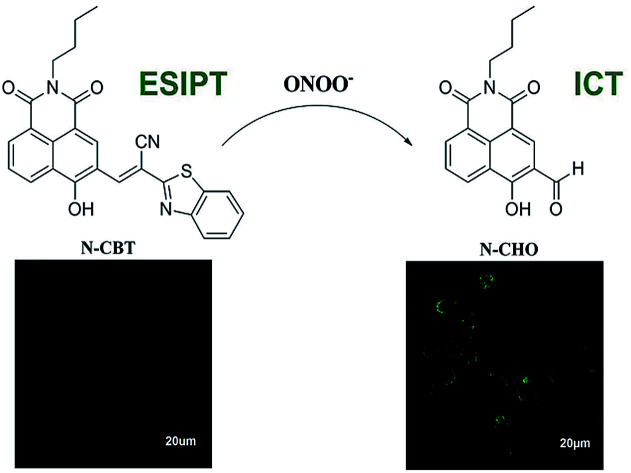

## 1. Introduction

Peroxynitrite (ONOO^−^) is one of the most important reactive oxygen species (ROS) in living systems generated by reaction of nitric oxide (NO) and superoxide radical anion (O_2_^−^˙).^1,2^ It features a relatively higher reactivity and thus shorter lifetime in physiological and pathological processes.^3–5^ Excessive ONOO^−^ will cause critical damage to cellular biomolecules, such as tyrosine residues or proteins containing thiols, DNA, and unsaturated fatty-acid-containing lipids by oxidation or nitrification, leading to diseases including cancer, diabetes, Alzheimer's disease, Parkinson's disease, Huntington's disease, inflammatory diseases, *etc.*^6,7^ Recent research^8–10^ has revealed that ONOO^−^ is also involved in cellular signal transduction as well as cell apoptosis. Thus, the monitoring of cellular peroxynitrite has captured lots of attention in order to elucidate its cell physiological function and pathological mechanism that lead to peroxynitrite-related disease diagnosis.

Among the methods available for ONOO^−^ detection, small molecule fluorescent probes have attracted much attention due to their manipulation simplicity, high selectivity, noninvasive nature and their widespread application in real time fluorescence imaging.^11–13^ The key strategy in the design of new and well-organized chemosensors is to employ proper signaling mechanisms.

Up to now, major achievements have been made in ONOO^−^ responsive fluorescence probe based on different photophysical processes.^14–27^ Conventional sensing mechanisms including photo-induced electron transfer (PET), intramolecular charge transfer (ICT), metal–ligand charge transfer (MLCT), twisted intramolecular charge transfer (TICT), electronic energy transfer (EET), fluorescence resonance energy transfer (FRET), in which PET, ICT and FRET have been used in peroxynitrite fluorescent probe,^28–40^ the lately reported ONOO^−^ probes are summarized in Table S1.† For example, Fabiao Yu *et al.*^39,40^ selected heptamethine cyanine dye with NIR fluorescence as the fluorophore, and used 4-(phenylselenyl)aniline and 2-(phenyltellanyl)benzohydrazide as fluorescence modulator respectively, to construct two ONOO^−^ fluorescent probes based on PET fluorescence switching mechanism. Based on ICT sensing mechanisms, Tang's group^32^ constructed a new ONOO^−^ probe (TP-KA) by installing α-ketoamide onto a fluorophore,1,8-naphthalimide. X. Zhou *et al.*^36^ developed a ratiometric and colorimetric probe CHCN, which contains a hybrid coumarin–hemicyanine scaffold. Xiaotong Jia *et al.*^33^ developed a probe (PNCy3Cy5) based on modulating FRET between Cy3 and Cy5 for ratiometric detection of ONOO^−^. Subsequently, Dan Cheng *et al.*^31^ presented a new two-photon ratiometric fluorescent ONOO^−^ probe (MITO-CC) with coumarin fluorophore based on the same mechanism. Excited state intramolecular proton transfer (ESIPT), another photophysical process has been attracting considerable interest since the past few years in the field of chemosensors because of their desirable unique photophysical properties. In generally, when ESIPT takes place, it implies low quantum yield (low background), which is exploited by some fluorescent probes.^41–47^ Recently, Li *et al.*^48^ developed a high sensitivity and two-photon excitable green fluorescent probe with the emission wavelength of 470 nm that designed by blocking the ESIPT process of the 2-(2′-hydroxyphenyl)benzothiazole fluorophore, for tracking the *in situ* generation of ONOO^−^ in cells and mice suffering from brain microvessel injury. Based on the above design of blocking the ESIPT process, Adam C. Sedgwick *et al.*^49^ incorporated benzyl boronic ester with the fluorescent *N*-methyl-benzothiazole gcore to develop an ONOO^−^ probe with an emission wavelength of 460 nm and successfully applied it to the detection of exogenous and endogenous peroxynitrite in living cells. However, the sensitivity of this probe was relatively low, which makes it not the best choice for bio-imaging. Also, probes with larger stocks shift and longer absorption/emission wavelength are desired.

To achieve this goal, we present an ESIPT-based small-molecule fluorescent probe (N-CBT) for visualizing endo/exogenous ONOO^−^ in living cells. It is composed of 3-formyl-4-hydroxy-1,8-naphthalic-*N*-butylimide (N–CHO) and benzothiazole linked through a cyano substituted carbon–carbon double bond. It is found to be highly selective towards ONOO^−^ in the scope of other ROSs and RNSs. The detection limit is calculated to be 37 nM. Moreover, we successfully applied N-CBT to monitor endo/exogenous ONOO^−^ in living cells.

## 2. Materials and methods

### 2.1 Reagents and apparatuses

All the chemicals were provided by commercial companies and used without further purification in this paper. The ^1^H NMR and ^13^C NMR spectra were measured with a Bruker Avance III at 400 MHz and 100 MHz respectively, and the chemical shifts were reported as ppm (in DMSO-*d*_6_ and CDCl_3_, TMS as internal standard). Mass spectra (MS) were determined by a Bruker Apex IV FTMS with the electrospray ionization. Absorption and fluorescence spectra were acquired by a Purkinje TU-1901 spectrophotometer and a Hitachi F-7000 fluorescence spectrometer. A pH acidometer (Mettler Toledo FE-30) was used for pH measurements. HPLC chromatogram was measured by an HPLC system consisted of two pumps (LC-20AT, SHIMADZU) and a UV detector (SPD-M10Avp, SHIMADZU), and an inertsil C18-ST (4.6 × 250 mm) column (Tech-Mate Technology Co., Ltd) was used.

### 2.2 The synthesis of N-CBT

The N–CHO (297 mg, 1 mmol) was dissolved in ethanol (15 mL) completely, 2-benzothiazoleacetonitrile (174 mg, 1 mmol) and triethylamine (0.1 mL) were added to the above solution, the mixture was stirred and refluxed at 78 °C overnight under nitrogen atmosphere. After cooling and vacuum filtration, the solids were washed by ethanol twice, and finally N-CBT triethylamine salt as a dark red crystal (340 mg, yield 75%) was recovered after vacuum drying.

#### N–CHO

^1^H NMR (400 MHz, CDCl_3_): *δ* 1.01 (t, 3H), 1.48 (q, 2H), 1.74 (m, 2H), 4.21 (m, 2H), 7.84 (t, 1H), 8.77 (m, 3H), 10.15 (s, 1H). ^13^C NMR (100 MHz, CDCl_3_): *δ* 13.84, 30.36, 30.19, 40.30, 114.88, 115.15, 122.82, 127.02, 130.30, 131.75, 134.09, 134.85, 163.00, 163.66, 165.67, 196.43. HR ESI-MS calcd for C_17_H_14_NO_4_^−^ [M − H]^−^: 296.0928, found 296.0932.

#### N-CBT

^1^H NMR (400 MHz, DMSO-*d*_6_) *δ* 9.32 (s, 1H), 8.81 (s, 1H), 8.47 (dd, *J* = 7.8, 1.3 Hz, 1H), 8.31 (dd, *J* = 7.5, 1.3 Hz, 1H), 8.08 (d, *J* = 7.9 Hz, 1H), 7.96 (d, *J* = 8.1 Hz, 1H), 7.50 (dt, *J* = 9.5, 7.1 Hz, 2H), 7.40 (t, *J* = 7.6 Hz, 1H), 4.02 (t, *J* = 7.4 Hz, 2H), 1.59 (h, *J* = 6.5, 5.8 Hz, 2H), 1.35 (q, *J* = 7.4 Hz, 2H), 0.94 (t, *J* = 7.3 Hz, 3H).^13^C NMR (101 MHz, DMSO-*d*_6_) *δ* 177.58, 166.96, 164.33, 154.01, 144.62, 134.28, 133.97, 133.13, 132.29, 131.55, 129.39, 127.08, 125.24, 124.16, 122.47, 122.41, 122.00, 119.01, 116.20, 102.82, 92.45, 49.07, 39.79, 30.41, 20.39, 14.27. HR ESI-MS calcd for C_26_H_20_N_3_O_3_S^+^ [M + H]^+^: 454.1220, found 454.1253.

### 2.3 The preparation of various analytes

A stock solution of N-CBT (5.0 mM) was prepared in dimethyl sulfoxide (DMSO). The working standard solutions were prepared by diluting the stock solution to 5 μM by ethanol: ultrapure water (5 : 5, v/v) containing phosphate buffered saline (PBS, 5 mM, pH = 7.4).

The sodium hypochlorite (NaClO) was prepared by diluting the 11–14% solution, hydrogen peroxide (H_2_O_2_) was prepared by diluting the 35% (w/w) solution, HNO was prepared by the Angeli's salt (AS), *tert*-butyl hydroperoxide (TBHP) was prepared by diluting the 70% solution, sodium nitroprusside was irradiated with ultraviolet light to get nitric oxide (NO), singlet oxygen (^1^O_2_) was prepared by the reaction between H_2_O_2_ and NaClO, hydroxyl radical (OH˙) was prepared by the reaction between iron(II) perchlorate hydrate and hydrogen peroxide, and glutathione (GSH) and cysteine (Cys) was dissolved into the distilled water.

ONOO^−^ is prepared by the reaction of NaNO_2_ and H_2_O_2_ under acid condition and stored in alkaline solution, and its concentration was determined by UV-vis spectrophotometer, according to the equation: *A* = *εbc* (*ε* was 1670 M^−1^ cm^−1^ at 302 nm).

### 2.4 Cell cultures

HeLa cells and macrophages were grown on glass-bottom culture dishes using Dulbecco's modified eagle media (DMEM) supplemented with 10% (v/v) fetal bovine serum (FBS) and 100 μg mL^−1^ penicillin–streptomycin in a humidified 37 °C, 5% CO_2_ incubator. For the detection of exogenous ONOO^−^, the HeLa cells were incubated with N-CBT (5 μM) for 30 min at 37 °C and washed with PBS buffer three times, and then added fresh prepared ONOO^−^ (20 μM). For the detection of endogenously produced ONOO^−^, the macrophages stimulated with 1 μg mL^−1^ lipopolysaccharides (LPS) and 50 ng mL^−1^ interferon-gamma (IFN-γ) for 12 h, and then 10 nM phorbol 12-myristate 13-acetate (PMA) for 30 min before incubated with 5 μM N-CBT for 30 min, and then washed with PBS buffer three times. The cells were observed by OLYMPUS FV-1000 inverted fluorescence microscope with 60× objective lens, and the excitation wavelength was selected as 405 nm.

## 3. Results and discussion

### 3.1 N-CBT design and synthesis

To construct an ESIPT chemosensor for selective detection of ONOO^−^, we employ the fluorescent naphthalimide, one of the most promising fluorophores, as the scaffold, and conjugate with 2-benzothiazoleacetonitrile. As shown in Scheme S1,† N–CHO was concentrated with equivalent 2-benzothiazoleacetonitrile in ethanol in the presence of triethylamine for 12 hours to yield final product, N-CBT. Different form previously reported naphthalimide chemosensors for which the silence of fluorescence is mostly caused by the cis–trans isomerization of the exocyclic C=C double bonds, in the case of N-CBT, the strong intermolecular hydrogen bond suppresses the rotation of aryl-alkene. Once N-CBT undergoes an ultrafast photon transfer in aqueous solution, its fluorescence will be quenched, and immediately followed by the perturbation of the ESIPT process upon interaction with ONOO^−^, yielding proper green fluorescence enhancement.

### 3.2 Photophysical properties of N-CBT

**Fig. 1:**
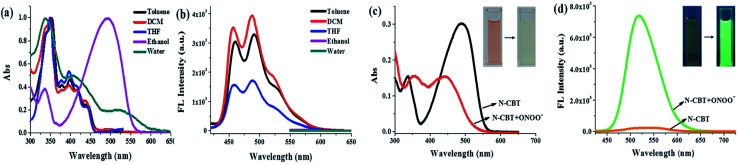
The normalized UV-vis absorption (a), and fluorescence emission (b) spectra of N-CBT (5 μM) in various solvents. *λ*_ex_ = 350 nm (toluene, tetrahydrofuran and dichloromethane); *λ*_ex_ = 530 nm (ethanol and water), slit widths are set as 5.0 nm. The UV-vis absorption (c), and fluorescence emission (d) spectra of N-CBT (5 μM) before and after the addition of ONOO^−^ (10 equiv.) in a mixture of ethanol and PBS (5 : 5, v/v, pH 7.4). Insets: (c) pictures of N-CBT before (left) and after (right) the addition of 10 equivalents of ONOO^−^, (d) photos of N-CBT before (left) and after (right) the addition of 10 equivalents of ONOO^−^ in a mixture of ethanol and PBS (5 : 5, v/v, pH 7.4) under the irradiation of 365 nm light; *λ*_ex_ = 405 nm, slit widths are set at 5.0 nm.

First, the photophysical properties of probe N-CBT were investigated by spectrophotometer and fluorescence spectrometer. The UV-vis absorption spectra of N-CBT in different solvents were shown in Fig. 1a. The absorption peak appeared at 350 nm and 400 nm in aprotic solvents (toluene, tetrahydrofuran and dichloromethane), and the absorption peak at 400 nm red shifted when in protic solvents such as ethanol and water. This probably is due to the tautomerization of N-CBT from alcohol to ketone, suggesting an ESIPT process from the phenolic proton (O–H) to the carbonyl oxygen. The fluorescence emission spectra of N-CBT in aprotic solvents (Fig. 1b) show two peaks at 460 nm and 500 nm, respectively. While in protic solvents, N-CBT showed barely any fluorescence even under the excitation at 530 nm light source. This result is accordant with fact that ESIPT sensor is usually sensitive to protic solvents, in which its emission will be inhibited to a great extent. Subsequently, we have also initiated fluorescence lifetime studies employing a nanosecond single-photon-counting spectrofluorometer, using nitrogen flash-lamp excitation and data analysis procedures as in routing studies. Two lifetimes were observed. Preliminary data indicated a biexponential decay kinetics with lifetimes of 2.6 ns and 0.9 ns in DCM at 298 K (Fig. S1†).

### 3.3 Reaction between N-CBT and ONOO^−^

To get a better understanding of the reaction between N-CBT and ONOO^−^, fresh prepared ONOO^−^ solution was titrated into N-CBT in the mix solution of PBS and ethanol. Upon the addition of ONOO^−^ (0–10 equiv.), the absorption intensity of N-CBT at 336 and 490 nm decreased, while the absorption band at 356 nm and 442 nm increased (Fig. 1c). Contemporary, the color of the solution changed from orange to pale yellow, which can be easily observed by the naked eyes. As shown in Fig. 1d, the fluorescence intensity at 518 nm exhibited a nearly 34-fold enhancement upon addition of ONOO^−^ (10 equiv.). And the color of solution changed from light yellow to green under the irradiation of portable light under 365 nm.

### 3.4 Sensitivity assay

**Fig. 2:**
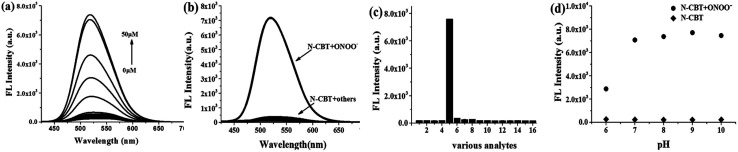
(a) The fluorescence responses of N-CBT (5 μM) toward different concentrations of ONOO^−^ from 0 to 50 μM, the solvent is a mixture of ethanol and PBS (5 : 5, v/v, pH 7.4); (b) and (c) fluorescence response of N-CBT (5 μM) toward ROSs, RNSs, bio-thiols and metal ions (the final concentration: GSH and H_2_O_2_ were 1 mM, Cys, Hcy and metal ions were 500 μM, the others were 50 μM), 1: blank; 2: Cys (500 μM); 3: Hcy (500 μM); 4: GSH (1 mM); 5: ONOO^−^ (50 μM); 6: H_2_O_2_ (1 mM); 7: TBHP (50 μM); 8: ClO^−^ (50 μM); 9: ^1^O_2_ (50 μM); 10: OH˙ (50 μM); 11: NO (50 μM); 12: HNO (50 μM); 13: Ca^2+^(500 μM); 14: Fe^2+^(500 μM); 15: Fe^3+^ (500 μM); 16: K^+^ (500 μM); 17: Mg^2+^ (500 μM); 18: Na^+^ (500 μM), the solvent is a mixture of ethanol and PBS (5 : 5, v/v, pH 7.4); (d) the effect of pH on the fluorescence intensity of N-CBT (5 μM) and N-CBT (5 μM) with ONOO^−^ (50 μM), the solvent is a mixture of ethanol and PBS (5 : 5, v/v, pH = 6, 7, 8, 9, 10). All data represent the fluorescence intensity at 518 nm, *λ*_ex_ = 405 nm, slit widths are set at 5.0 nm, cuvette width is 1 cm.

Fluorescence titration assay were employed to get a deep insight of this reaction in the aspect of sensitivity. In Fig. 2a, when ONOO^−^ concentration increased from 0 equiv. to 10 equiv., the fluorescence intensity of the solution increased gradually and finally reached a plateau at 34 fold. A good linear relationship between the fluorescence intensity at 518 nm and ONOO^−^ concentration (1–14 μM) was shown in Fig. S2b,† according to which, the detection limit was calculated to be 37 nM. In a similar way, under higher concentrations of ONOO^−^, fluorescence intensity also submits to a good linear relationship towards ONOO^−^ by applying more concentrated N-CBT (Fig. S2d†).Perfect segmentation fitting indicated a wide range of detection concentration, which is highly praised in cellular ONOO^−^ monitoring.

### 3.5 Selectively studies

Selectivity is one of the important properties for the probe, so we evaluated the selectivity of N-CBT towards ONOO^−^ in the presence of other cellular interferents. The fluorescence spectra of the probe with other ROSs (H_2_O_2_, TBHP, ClO^−^, ^1^O_2_ and OH˙), RNSs (NO, HNO), bio-thiols (Cys, GSH and Hcy) and metal ions (Ca^2+^, Fe^2+^, Fe^3+^, K^+^, Mg^2+^, Na^+^) were measured. As shown in Fig. 2b and c, when H_2_O_2_, TBHP and ClO^−^ existed, the fluorescence intensity at 518 nm increased slightly, but compared with the increased fluorescence intensity of N-CBT with ONOO^−^, it can be ignored. And the fluorescence intensity of N-CBT with others has not obviously changed. Therefore, N-CBT exhibits high selectivity for ONOO^−^ determination, which indicated that the probe is capable of detecting ONOO^−^ in a complex physiological environment.

### 3.6 pH effect

The pH effect on fluorescence intensity of N-CBT was also tested. As shown in Fig. 2d, the fluorescence intensity of N-CBT kept extremely low in the pH range of 6–10. After adding 50 μM ONOO^−^, the fluorescence intensity in the pH range of 7–10 was intensified appreciably to 34-fold and stayed constant. However, when the pH is below 6, the increase in fluorescence intensity of N-CBT with ONOO^−^ is 12-fold, due to the fact that ONOO^−^ is more easily decomposed under acidic conditions.^50,51^ Therefore, the result indicates that N-CBT can be used to detect ONOO^−^ under physiological conditions. The dynamic experiments were performed under 37 °C at pH 7.4 to mimic the internal biological environment, the reaction time curve of N-CBT to ONOO^−^ obtained as shown in Fig. S3.†

### 3.7 Proposed sensing mechanism

**Scheme 1:**
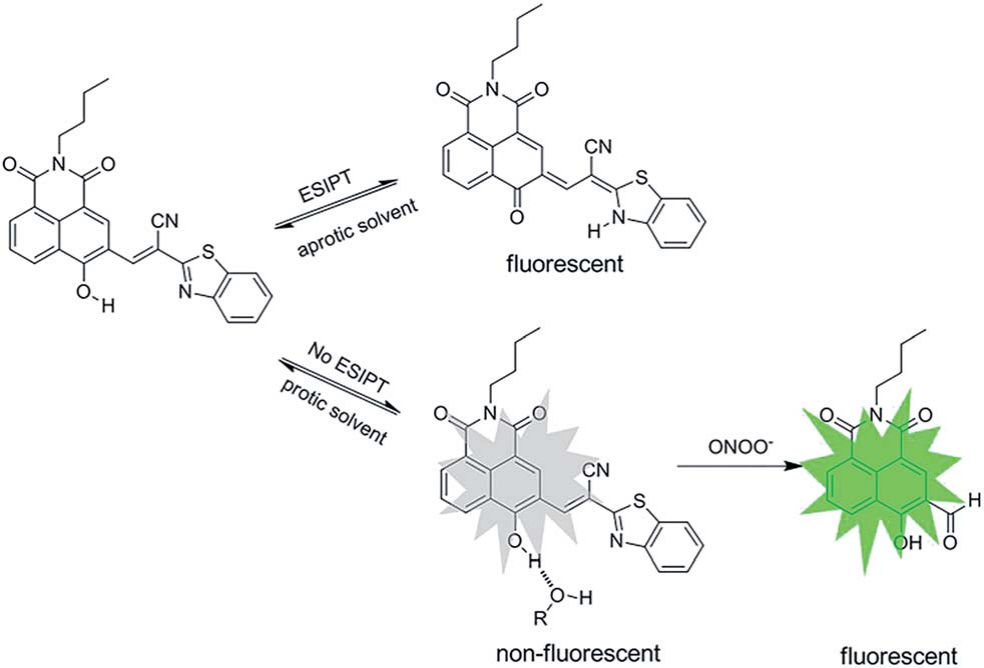
Proposed sensing mechanism of N-CBT with ONOO^−^.

The reaction was further confirmed by high performance liquid chromatography (HPLC), ^1^H NMR and HRMS. As we can see from Fig. S4,† the retention time of product generated from N-CBT with ONOO^−^ is in agreement with that of its parent fluorophore N–CHO. From the ^1^H-NMR spectra (Fig. S5†), we noticed the disappearance of aromatic protons belonging to N-CBT, and the generation of N–CHO especially the gradually arising of characteristic hydrogen of aldehyde around 10.3 ppm. From the high-resolution mass spectrometry (Fig. S6†), a main peak at [M − H]^−^ = 296.0929 is obtained, which is coincide with the theoretical calculation data of N–CHO ([M − H]^−^ = 296.0928). Subsequently, we measured the fluorescence decay curves of the reaction mixture of N-CBT with ONOO^−^, the results are shown in the Fig S7.† A single exponential decay with average lifetime of the reaction mixture in DCM is 4.0 ns, which is identical to a single exponential decay with calculated lifetime of N–CHO (3.7 ns) (Fig. S7†). While N-CBT showed biexponential decay kinetics with lifetimes of 2.6 ns and 0.9 ns in DCM. Therefore, these experimental results indicated that N-CBT transformed into N–CHO after the reaction with ONOO^−^. Thus, we rationally speculate that, the ONOO^−^ acts as a nucleophile to attack the cyano-substituted electron-withdrawing C=C bond, resulting in the release of N–CHO, as shown in Scheme 1.

### 3.8 Computed results

**Fig. 3:**
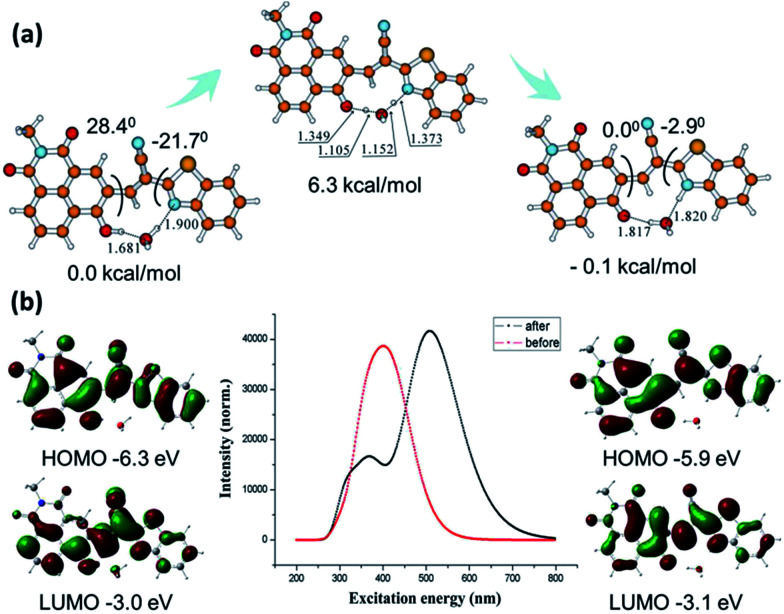
Computational results. (a) Optimized structure and bond distances conformations of N-CBT. These figures were obtained from Gaussian09. (b) Representation of the UV-visible absorption and emission of N-CBT using TD-DFT. The vertical excitation related calculations were based on optimized ground state geometry and the emission based calculations were based on optimized excited state geometry at B3LYP/6-31G(d).

Both enol form and keto form of N-CBT involved during the proton transfer are illustrated in Fig. 3a. All electronic structure computations were carried out using the Gaussian 09 program. The ground state (S_0_) geometry of tautomers of N-CBT were optimized using the tight criteria in the gas phase using Density Functional Theory (DFT). The functional used was B3LYP. The basis set used for all atoms was 6-31G(d). The vibrational frequencies at the optimized structures were computed using the same method to verify that the optimized structures correspond to local minima on the energy surface. In the presence of water, the O(C=O)–H(OH) bond distances and angles between two planes in the ground state were optimized and given. The stale intercellular H bond and increasing molecular planarity which is the major factor necessary for ESIPT facilitating the proton transfer in the excited state reveals the facility of keto–enol transformation in ground state. The cyclic four-level photochemical and photophysical process of phototautomerizable N-CBT involving ESIPT (K_enol_ → K_enol^*^_ → K_keto^*^_ → K_keto_) is exemplified in chloroform. Frequency computations were also carried out on the optimized geometry of the low-lying vibronically relaxation of the first excited state for enol form and keto form. The vertical excitation energies at the ground state equilibrium geometries were calculated with Time-Dependent Density Functional Theory TD-DFT [B3LYP/6-31G(d)]. The HOMO and LUMO energy levels in the excited states are greatly different from those in the ground states. The gaps between the energies of the optimized geometries at the first singlet excited state and the ground state was used to predict the absorption and emissions. As shown in Fig. 3b, the computed absorption band is at 400 nm, while the emission peak is around 530 nm. Experimental absorption and emission wavelengths are in good agreement with those predicted results. Since ESIPT is largely characterized by the energy level changes of K_enol^*^_ → K_keto^*^_, the 0.4 eV ignorable energy barrier from −6.3 eV to −5.9 eV is allowed. This result further validates the efficient ESIPT process.

### 3.9 ONOO^−^ monitoring in living cells

**Fig. 4:**
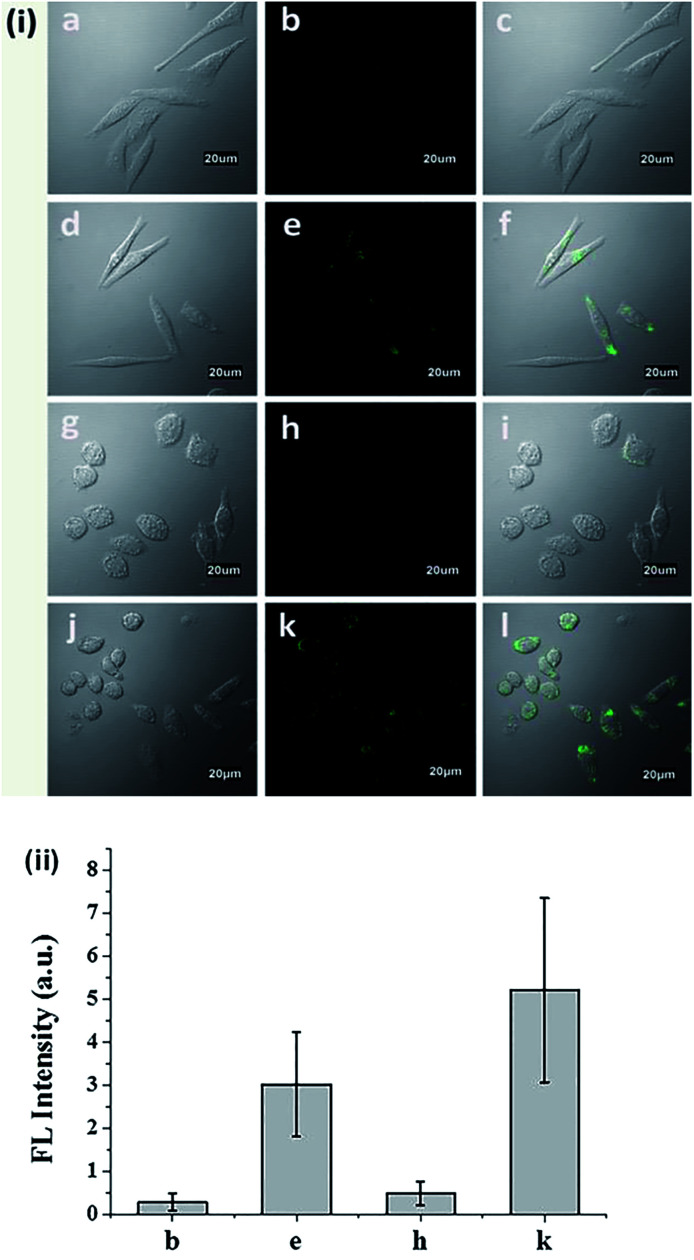
(i) Bright field (left), fluorescence (middle) and overlay (right) images of (a–c) HeLa cells treated with 5 μM N-CBT for 30 min, (d–f) HeLa cells treated with 5 μM N-CBT for 30 min followed by the addition of 20 μM ONOO^−^, (g–i) macrophages cells treated with 5 μM N-CBT for 30 min, (j–l) macrophages stimulated with 1 μg mL^−1^ LPS and 50 ng mL^−1^ IFN-γ for 12 h, and then 10 nM PMA for 30 min before incubated with 5 μM N-CBT for 30 min. (ii) Fluorescence intensity of fluorescence images b, e, h and k, the results are presented as mean ± standard deviation (*n* ≥ 8).

The cytotoxicity of N-CBT was evaluated before the cell imaging experiment. From Fig. S8† we can see, with the increase of N-CBT concentration from 0 to 25 μM, the HeLa cells survival rate was decreased from 100% to 70%, and macrophage survival rate of 85% or more. It's easy to follow that N-CBT showed hardly any cytotoxicity to HeLa cells and macrophages under the working concentration, 5 μM. The ability of N-CBT for cellular detection was used to detect ONOO^−^ in HeLa cells and macrophages, and the results of cell imaging were shown in Fig. 4. As can be observed from Fig. 4i(a–f),the HeLa cells showed extremely low fluorescence after incubated with 5 μM probe for 30 minutes without any stimulation. After adding 20 μM ONOO^−^, strong cellular green fluorescence was observed. Macrophages were stimulation with LPS, IFN-ϒ and PMA to produce endogenous ONOO^−^, the results are shown in the Fig. 4i(g–l). As we have seen, the macrophages that were not stimulated were barely fluorescent after incubated with 5 μM probe for 30 minutes. However, the stimulated macrophages had significant green fluorescence. The fluorescence of cells was measured, and the results shown in Fig. 4ii demonstrated that the HeLa cell and macrophages has 44 fold and 45 fold fluorescence enhancement, respectively. These results indicated that N-CBT features good cell membrane permeability and specific ability to detect endogenous and exogenous ONOO^−^ in living cells.

## 4. Conclusions

In summary, we designed and synthesized a chemosensor N-CBT based on ESIPT mechanism. By incorporation of 2-benzothiazoleacetonitrile, N-CBT shows a rare pre-existing eight-membered ring hydrogen bonding configuration, which is able to generate H bond sensitive dual emissions. In aqueous solution, the formation of intermolecular hydrogen bonding with water destroys the stable planar construction and gives an off fluorescence state. By reaction with ONOO^−^, green fluorescence at 518 nm can be regenerated efficiently. The results showed a good linear relationship in the range of 1 to 14 μM with a detection limit of 37 nM. Benefiting from its high sensitivity and selectivity, N-CBT was successfully applied in visualizing cellular ONOO^−^, and it showed a great potential in selectively visualizing endo/exogenous peroxynitrite (ONOO^−^) in living cells.

## Conflicts of interest

There are no conflicts to declare.

## Supplementary Material

RA-008-C7RA11774D-s001
