# Supplementary material for: An ESIPT based naphthalimide chemosensor for visualizing endogenous ONOO− in living cells
Source: RSC Adv. 2018 Jan 8;8(4):1826–32. doi: 10.1039/c7ra11774d (PMC9077266; doi:10.1039/c7ra11774d)
Supplement: RA-008-C7RA11774D-s001 [file RA-008-C7RA11774D-s001.pdf]

Electronic Supplementary Material (ESI) for RSC Advances.
This journal is © The Royal Society of Chemistry 2018

Supporting Information for

An ESIPT based naphthalimide chemosensor for visualizing endogenous $ONOO^-$ in living cells

Yunshuang Fu, Hailiang Nie, Rubo Zhang, Fangyun Xin, Yong Tian, Jing Jing * and Xiaoling Zhang*

Key Laboratory of Cluster Science of Ministry of Education, Beijing Key Laboratory of Photoelectronic/Electrophotonic Conversion Materials, Analytical and Testing Center, School of Chemistry and Chemical Engineering, Beijing Institute of Technology, Beijing 100081, P. R. China

E-mail: zhangxl@bit.edu.cn (X. Zhang); Tel./fax: +86-010-88875298.

E-mail: hellojane@bit.edu.cn (J. Jing); Tel./fax: +86-010-81381351

Contents

1. **Determination of detection limit.**
2. **The summary table of lately $ONOO^-$ probe.**
3. **The synthesis route of probe N-CBT.**
4. **Determination of fluorescence lifetime.**
5. **Linear relationship between concentration of $ONOO^-$ and fluorescence intensity.**
6. **Reaction time of N-CBT to $ONOO^-$.**
7. **HPLC chromatogram of N-CBT, N-CHO and N-CBT with $ONOO^-$.**
8. **^{1}H-NMR spectrum comparison of N-CBT, N-CBT with $ONOO^-$ and N-CHO.**
9. **HR MS of the reaction mixture of N-CBT with $ONOO^-$.**
10. **Determination of fluorescence lifetime of N-CBT with $ONOO^-$ and N-CHO.**
11. **MTT assay.**
12. **The characterization data of N-CBT.**

1. Determination of detection limit

The detection limit (DL) was calculated by the fluorescence titration experiment, the fluorescence intensity of the blank sample was determined for ten times, the date were used to calculate the standard deviation. The detection limit was calculated with the following equation:

$$DL=3б/k$$

In the equation, б is the standard deviation of blank measurements; k is the slope of the fluorescence intensity versus the concentration plot.

2. The compared table of lately $ONOO^-$ probe

Table S1 Summary of lately reported fluorescent probes for $ONOO^-$.

probe	sensing mechanism	λex /λem	stokes shift	LOD
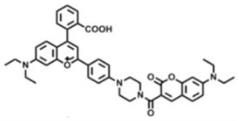 J. Am. Chem. Soc. 2017, 139, 285-292.	FRET	420 nm<br>473 nm/651nm	N.A	11.30 nM
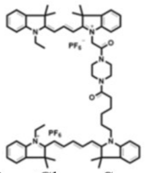 J. Am. Chem. Soc. 2016,138, 10778-10781.	FRET	530 nm<br>560 nm/660 nm	30nm<br>20nm	0.65 nM
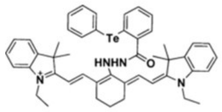 J. Am. Chem. Soc., 2013, 135, 7674-7680.	PET	793 nm<br>820 nm	27nm	0.917μM
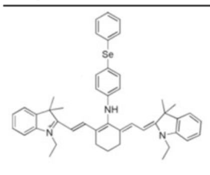 J. Am. Chem. Soc. 2011, 133, 11030-11033.	PET	758 nm<br>775 nm	17nm	N.A
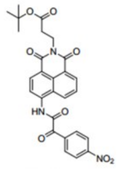 Chem. Sci. 2017, 8, 4006-4011.	ICT	430 nm<br>560 nm	130nm	25nM

probe	sensing mechanism	λex /λem	stokes shift	LOD
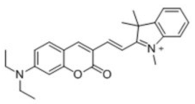 Biosens. Bioelectron., 2015,64, 285-291.	ICT	475 nm<br>515 nm/635 nm	N.A	49.7 nM
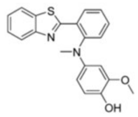 J. Am. Chem. Soc. 2015, 137, 12296-12303.	ESIPT	375 nm<br>470 nm	95nm	5.0 nM
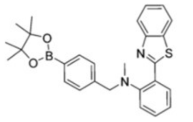 Chem. Commun. 2016, 52, 12350-12352.	ESIPT	400 nm<br>461 nm	61nm	N.A
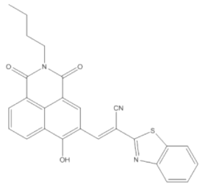 this work	ESIPT/ICT	405 nm<br>518 nm	103nm	37nM

3. The synthesis route of probe N-CBT

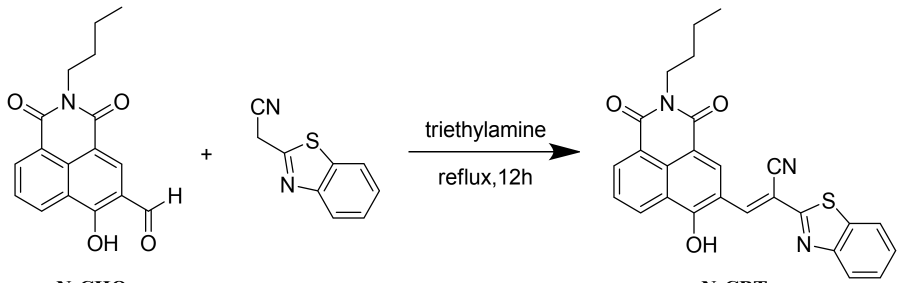

N-CHO **N-CBT**

Scheme S1 The synthesis route of probe N-CBT

4. Determination of fluorescence lifetime of N-CBT

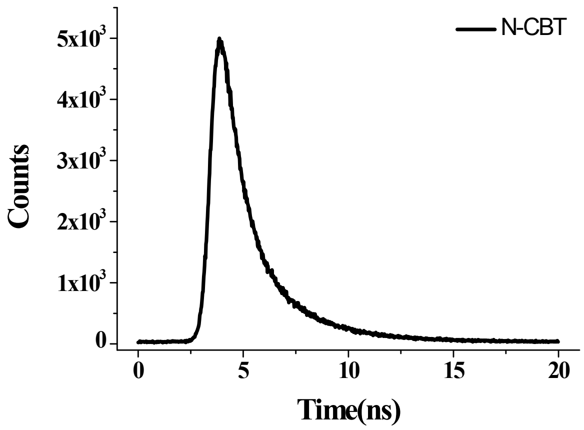

Fig S1 Fluorescence decay curves of N-CBT in DCM (λ_{ex}=350nm).

5. Linear relationship between concentration of $ONOO^-$ and fluorescence intensity.

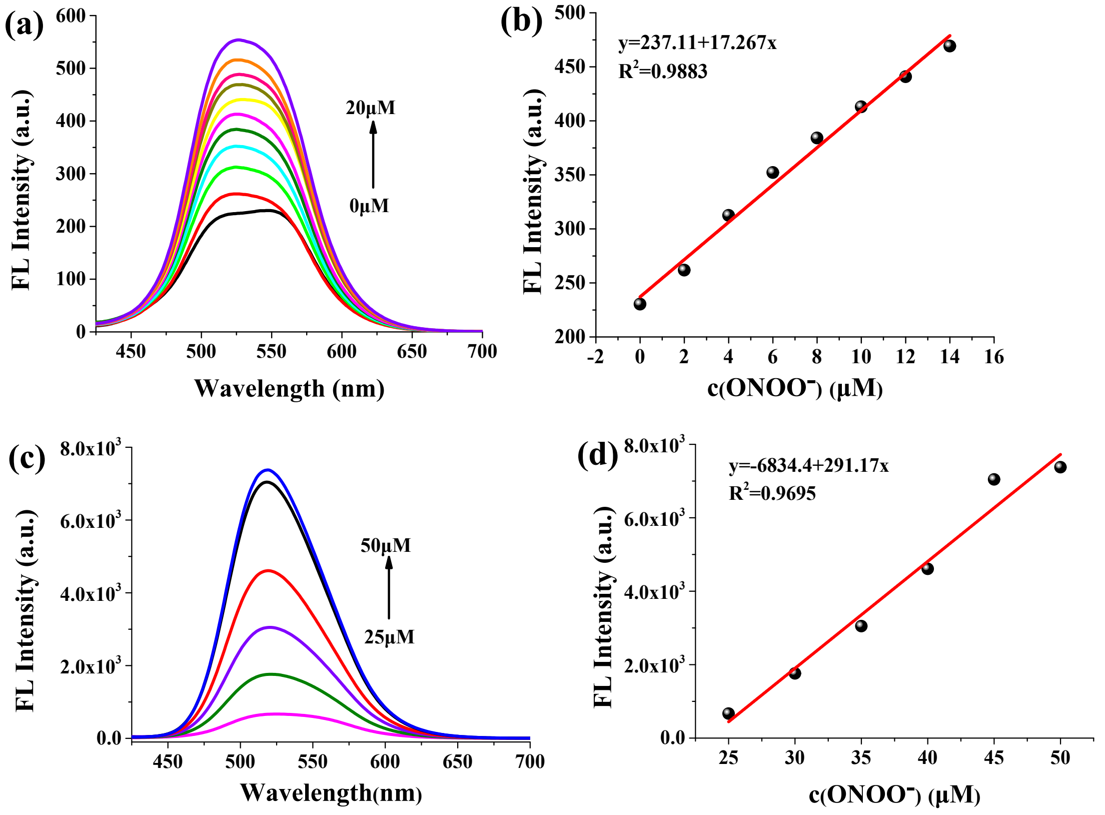

Fig S2 (a) The fluorescence responses of N-CBT (5 μM) toward different concentrations of $ONOO^-$ (0-20 μM). (b) The linear relationship between fluorescence intensity and $ONOO^-$ added in the range of 1-14μM; (c) The fluorescence responses of N-CBT (5 μM) toward different concentrations of $ONOO^-$ (25-50 μM). (d)The linear relationship between fluorescence intensity and $ONOO^-$ added in the range of 25-50 μM;the solvent is a mixture of ethanol and PBS (5:5, v/v, pH 7.4), λex =405 nm, slit widths are

set at 5.0 nm, cuvette width is 1cm.

6. Reaction time of N-CBT to $ONOO^-$.

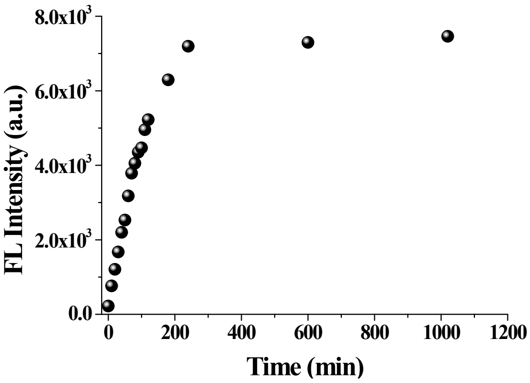

Fig S3 The fluorescence intensity of N-CBT (5 μM) after the addition of $ONOO^-$ (50 μM) after different reaction time in a mixture of ethanol and PBS (5:5, v/v, pH =7.4). λex =405 nm, slit widths are set at 5.0 nm.

7. HPLC chromatogram of N-CBT, N-CHO and N-CBT with $ONOO^-$.

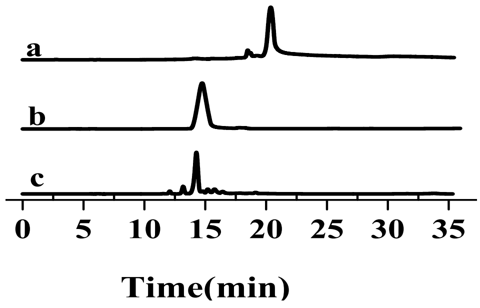

Fig S4 HPLC chromatogram of (a) N-CBT, (b) N-CHO and (c) N-CBT with ONOO-.

8. ^{1}H-NMR spectrum comparison of N-CBT, N-CBT with $ONOO^-$ and N-CHO

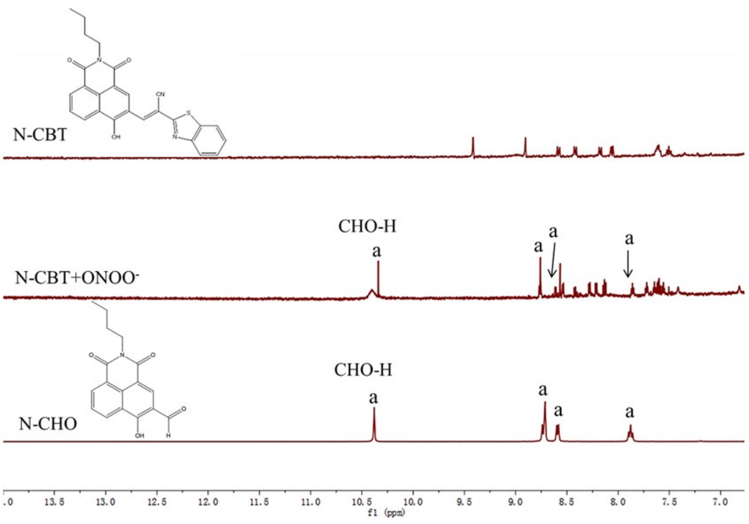

Fig S5 ^{1}H-NMR spectrum comparison of N-CBT, N-CBT with $ONOO^-$ and N-CHO in DMSO-d_6

9. HR MS of the reaction mixture of N-CBT with $ONOO^-$

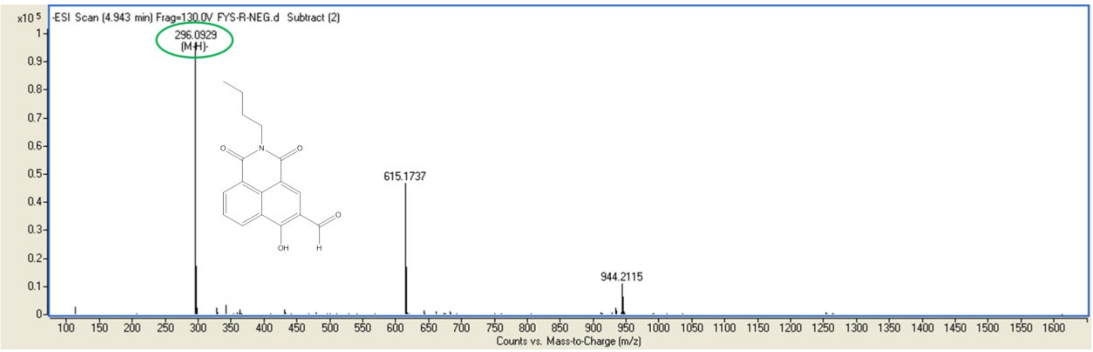

Fig S6 High resolution mass spectrum of N-CBT with $ONOO^-$

10. Determination of fluorescence lifetime of N-CBT with $ONOO^-$ and N-CHO

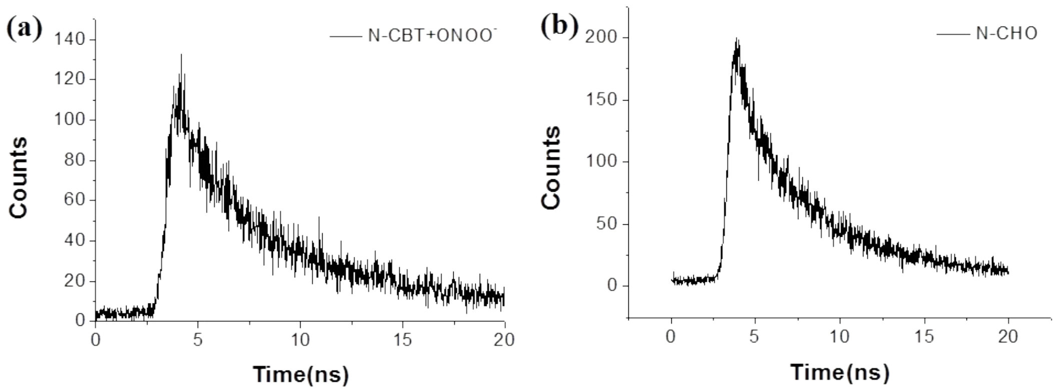

Fig S7 Fluorescence decay curves of (a)N-CBT with $ONOO^-$ and (b)N-CHO in DCM (λ_{ex}=350nm)

11. MTT assay

HeLa cells/macrophages were cultured in culture media (DMEM) in an atmosphere of 5% CO_2 and 95% air at 37 °C. The cells were seeded into 96-well plates at a density of 5 × 10^3 cells per well in culture media, then 0, 5, 10, 15, 20 and 25 μM N-CBT were added, respectively. Next, the cells were incubated at 37 °C in an atmosphere of 5% CO_2 and 95% air for 24 h. Finally, 10 μL 3-(4,5-dimethylthiazol-2-yl)-2,5-diphenyltetrazolium bromide (MTT, 5 mg/mL) was added and were cultured for another 3 h, respectively. Then 100μl DMSO added to each well and measured at 570nm at microplate reader (Bio-rad 680).

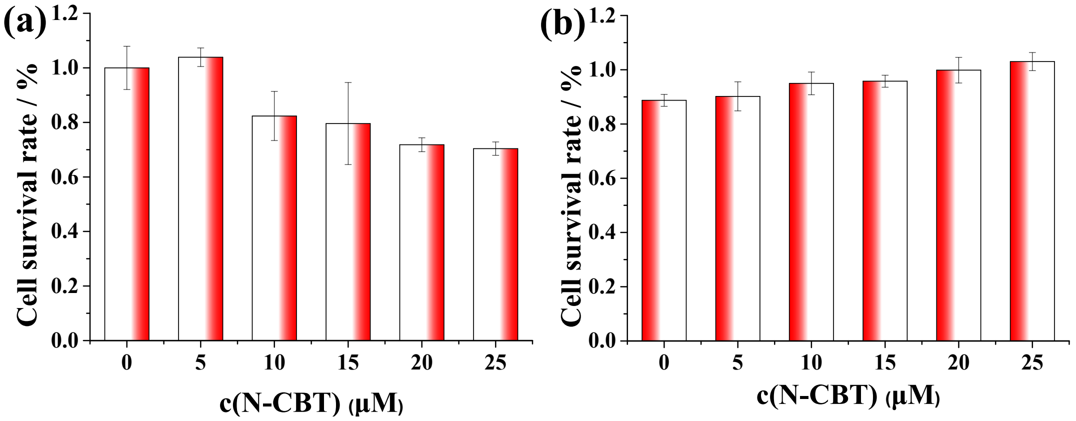

Fig S8 The cytotoxicity of different concentrations of the N-CBT (0, 5, 10, 15, 20 and 25 μM) in HeLa cells (a) and macrophages (b).

12. The characterization data of N-CBT.

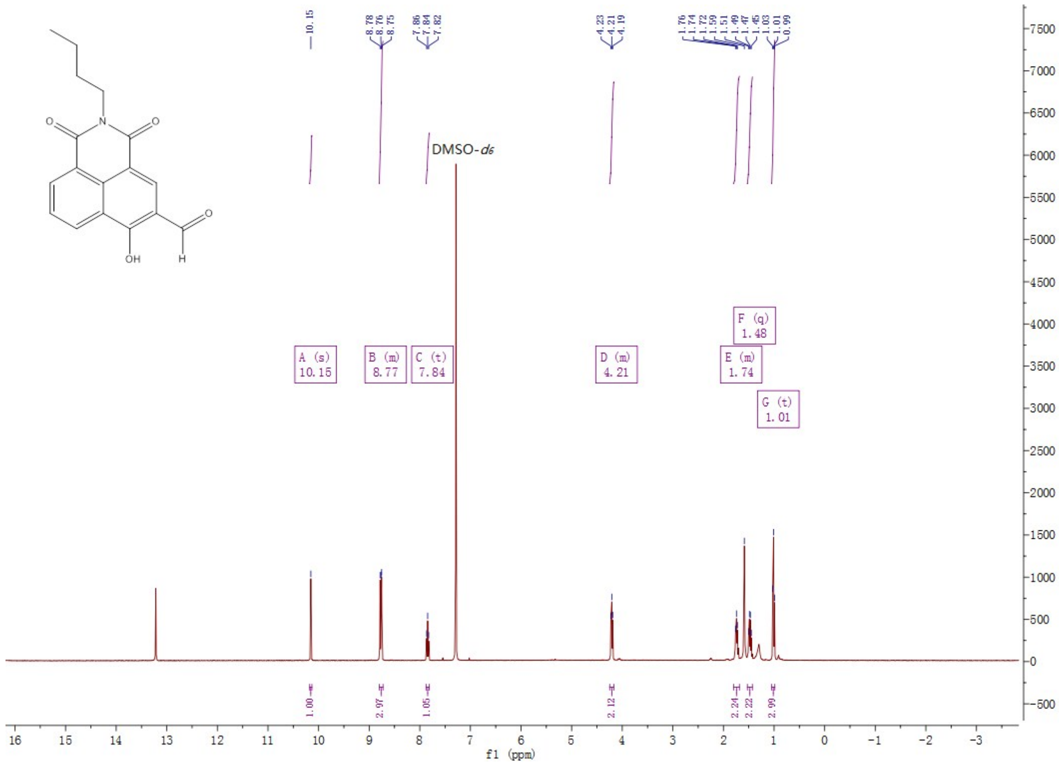

Figure S9 ^{1}H-NMR of N-CHO

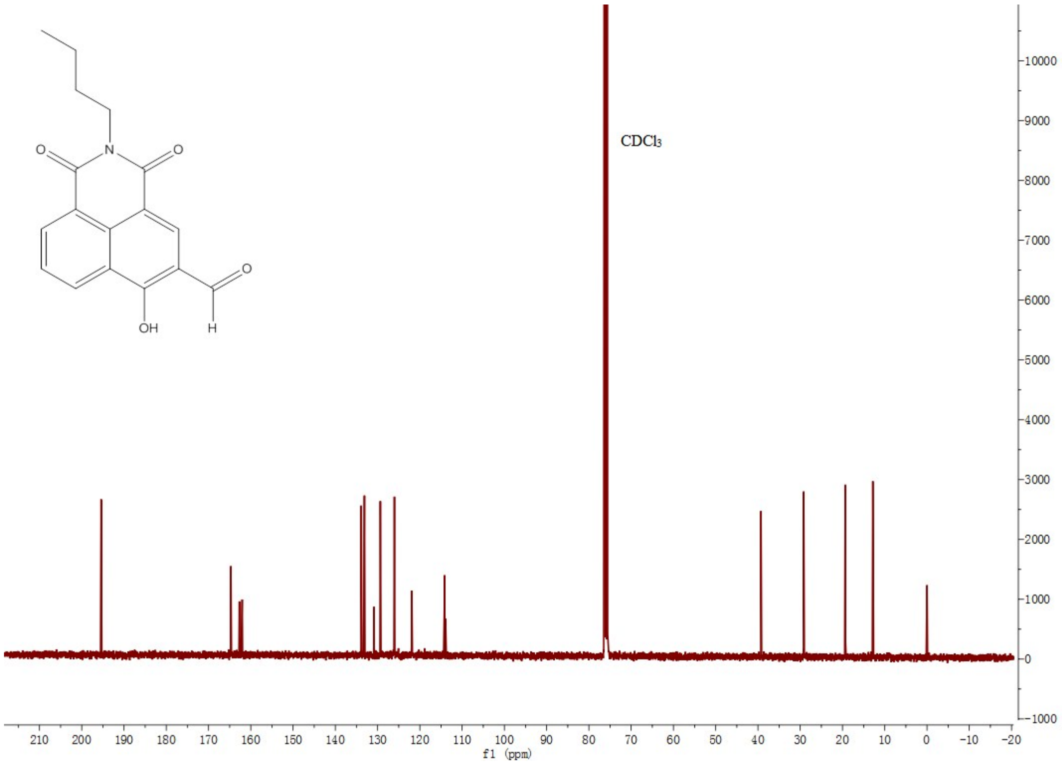

Figure S10 ^{13}C-NMR of N-CHO

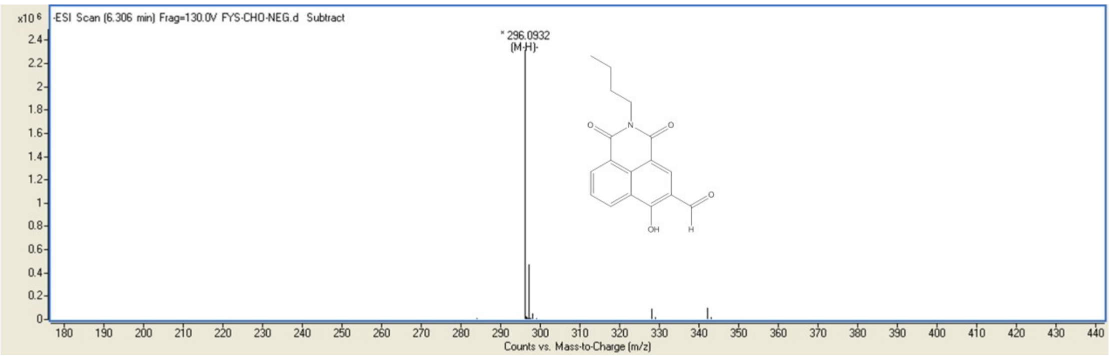

Figure S11 High resolution mass spectrum of N-CHO

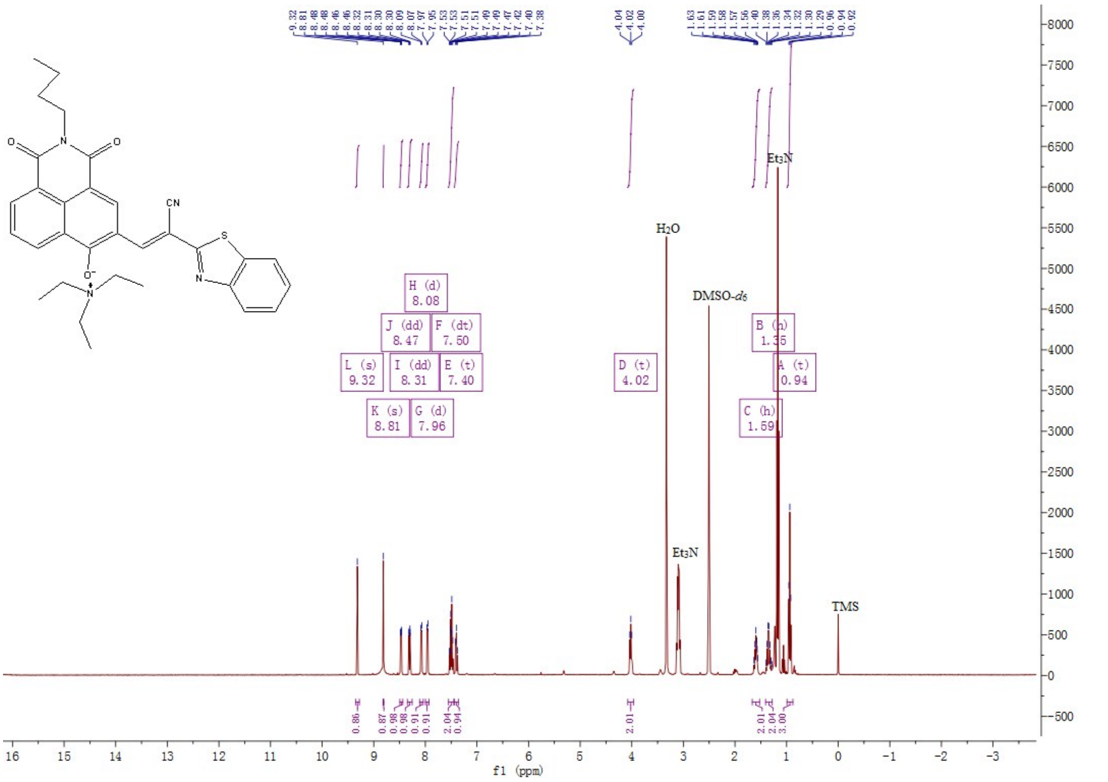

Figure S12 ^{1}H-NMR of N-CBT

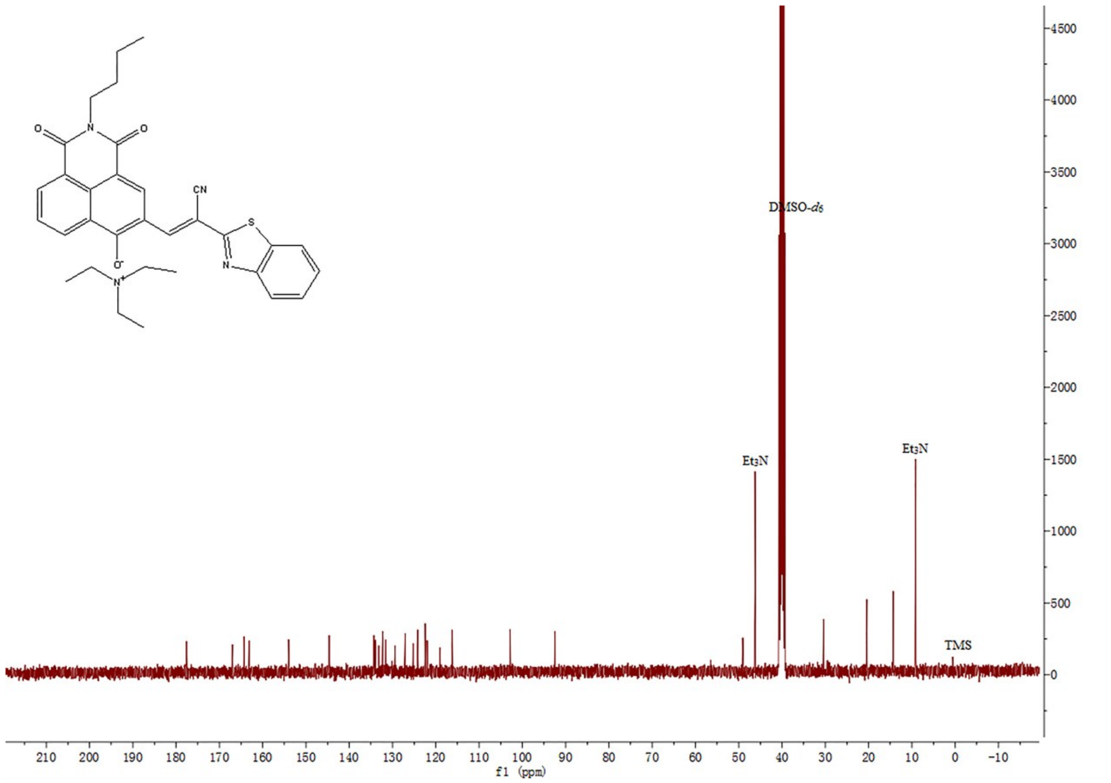

Figure S13 ^{13}C-NMR of N-CBT

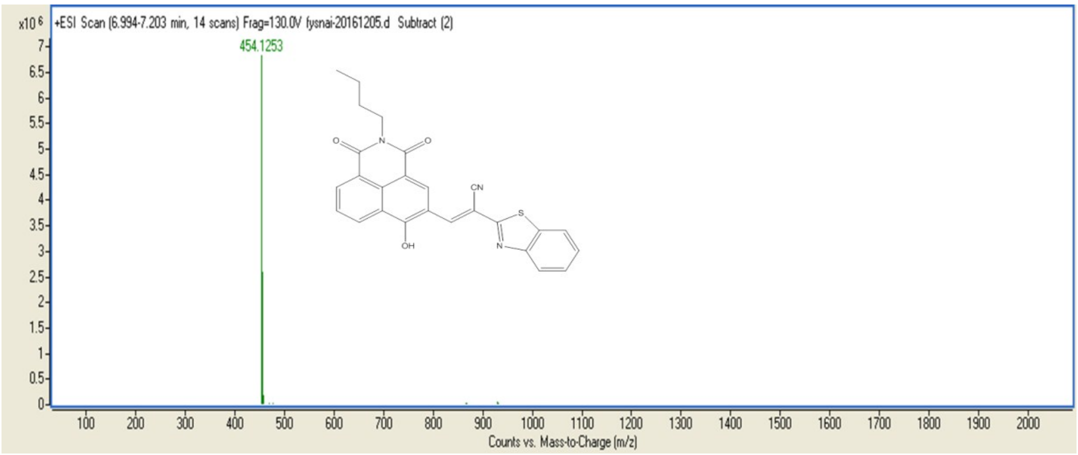

Figure S14 High resolution mass spectrum of N-CBT
